# The Tetraspanin Protein CD37 Regulates IgA Responses and Anti-Fungal Immunity

**DOI:** 10.1371/journal.ppat.1000338

**Published:** 2009-03-13

**Authors:** Annemiek B. van Spriel, Mariam Sofi, Kate H. Gartlan, Alie van der Schaaf, Ineke Verschueren, Ruurd Torensma, Reinier A. P. Raymakers, Bruce E. Loveland, Mihai G. Netea, Gosse J. Adema, Mark D. Wright, Carl G. Figdor

**Affiliations:** 1 Department of Tumor Immunology, Nijmegen Centre for Molecular Life Sciences, University Medical Centre, Nijmegen, The Netherlands; 2 Department of Immunology, Monash University, Alfred Medical Research and Education Precinct, Melbourne, Australia; 3 Department of Medicine and Nijmegen Institute for Infections, Inflammation and Immunity (N4i), University Medical Centre, Nijmegen, The Netherlands; 4 Department of Hematology and Central Hematology Laboratory, University Medical Centre, Nijmegen, The Netherlands; 5 Burnet Institute (Austin Campus), Heidelberg, Australia; David Geffen School of Medicine at University of California Los Angeles, United States of America

## Abstract

Immunoglobulin A (IgA) secretion by plasma cells in the immune system is critical for protecting the host from environmental and microbial infections. However, the molecular mechanisms underlying the generation of IgA^+^ plasma cells remain poorly understood. Here, we report that the B cell–expressed tetraspanin CD37 inhibits IgA immune responses *in vivo*. CD37-deficient (CD37−/−) mice exhibit a 15-fold increased level of IgA in serum and significantly elevated numbers of IgA^+^ plasma cells in spleen, mucosal-associated lymphoid tissue, as well as bone marrow. Analyses of bone marrow chimeric mice revealed that CD37–deficiency on B cells was directly responsible for the increased IgA production. We identified high local interleukin-6 (IL-6) production in germinal centers of CD37−/− mice after immunization. Notably, neutralizing IL-6 *in vivo* reversed the increased IgA response in CD37−/− mice. To demonstrate the importance of CD37—which can associate with the pattern-recognition receptor dectin-1—in immunity to infection, CD37−/− mice were exposed to *Candida albicans*. We report that CD37−/− mice are evidently better protected from infection than wild-type (WT) mice, which was accompanied by increased IL-6 levels and *C. albicans*–specific IgA antibodies. Importantly, adoptive transfer of CD37−/− serum mediated protection in WT mice and the underlying mechanism involved direct neutralization of fungal cells by IgA. Taken together, tetraspanin protein CD37 inhibits IgA responses and regulates the anti-fungal immune response.

## Author Summary

Antibody, or immunoglobulin (Ig), production by plasma cells in the immune system is important for protecting the host from microbial infections. IgA is the most abundant antibody isotype produced in the body. However, the molecular mechanisms underlying the generation of IgA–producing plasma cells remain poorly understood. We now report that the B cell–expressed protein CD37 regulates IgA immune responses, both in steady-state conditions and during infection. We found highly increased levels of IgA in serum and elevated numbers of IgA^+^ plasma cells in lymphoid tissue of mice that are deficient for CD37 (CD37−/− mice). To demonstrate the importance of CD37 in immunity to infection, CD37−/− mice were exposed to the fungus *Candida albicans*. *C. albicans* can cause systemic infection with high mortality in immunocompromised patients. We demonstrate that CD37−/− mice are evidently better protected from infection than wild-type mice, which was dependent on *C. albicans*–specific IgA antibodies. The underlying mechanism involved direct neutralization of fungal cells by IgA. In summary, the B cell protein CD37 inhibits IgA responses and anti-fungal immunity. This study may contribute to the development of novel immunotherapeutic approaches for invasive fungal disease.

## Introduction

Plasma cells (non-dividing antibody-secreting cells, ASC) are terminally differentiated B cells that are central to humoral immunity. Both mucosal and systemic immune responses to infection can induce IgA^+^ plasma cell formation, resulting in production of secretory and serum IgA respectively. In both pathways, isotype class-switching in B cells is tightly regulated by the cytokine-milieu. While it is believed that interleukin-6 (IL-6), originally described as a B-cell stimulating factor [1], stimulates plasma cell differentiation and promotes IgA responses *in vivo* [2], the molecular mechanisms underlying the development of IgA-secreting plasma cells remain poorly understood. In particular, detailed knowledge about proteins in the B cell membrane that control this process is lacking.

IgA-secreting plasma cells are predominantly found in the mucosal-associated lymphoid system (MALT) and bone marrow. During the mucosal immune response, intestinal dendritic cells present mucosal antigen to CD4^+^ T cells in Peyer's patches, or present antigen directly to B1 cells in a T cell-independent manner [3],[4]. This leads to the local production of secretory IgA, which is important in neutralizing intestinal microbes and controlling gut homeostasis. Secondly, systemic immune responses to T cell-dependent antigens lead to the development of germinal centers (GC) in spleen and peripheral lymph nodes that are the origin of long-lived plasma cells that produce high affinity IgG and IgA antibodies in serum [5]–[7]. Recent studies have underlined the importance of local production of B cell stimuli (cytokines, TLR ligands) in inducing IgA production during the humoral immune response [3],[8], including IL-6 that promotes the generation of IgA-secreting plasma cells [2],[3].

Tetraspanins, or transmembrane-four superfamily proteins, are implicated in organizing (immuno)-receptors, integrins, and signaling molecules into functional membrane complexes (tetraspanin microdomains) [9]–[12]. Consequently, tetraspanins are important in fundamental cellular processes including migration, proliferation, differentiation, and –when deregulated- cancer [13]. CD37 is expressed exclusively on cells of the immune system, in contrast to most other tetraspanins. CD37-deficient mice display defects in various arms of the immune system, including impaired T cell-dependent IgG responses, T cell hyperproliferation, and increased antigen-presenting capacity by dendritic cells [14]–[16]. We have recently demonstrated that the C-type lectin dectin-1 interacts with tetraspanin CD37 [17]. Dectin-1 recognizes β-glucans that are found in the cell wall of fungi, and is involved in cytokine production and killing of fungal pathogens including *Candida albicans* [18],[19].

In this study, we provide novel insights into the mechanisms underlying IgA production during the humoral immune response. We demonstrate that the B cell-expressed tetraspanin CD37 inhibits the formation of IgA-secreting plasma cells *in vivo* that is critically dependent on IL-6. Moreover, CD37-deficient mice are protected against *C. albicans* infection, which was dependent on fungal-specific IgA antibodies. Taken together, tetraspanin protein CD37 inhibits IgA responses both in steady state conditions and during infection. This is the first demonstration that tetraspanins control the immune-mediated defense against fungal pathogens.

## Results/Discussion

### CD37 inhibits IgA production *in vivo*

**Figure 1:**
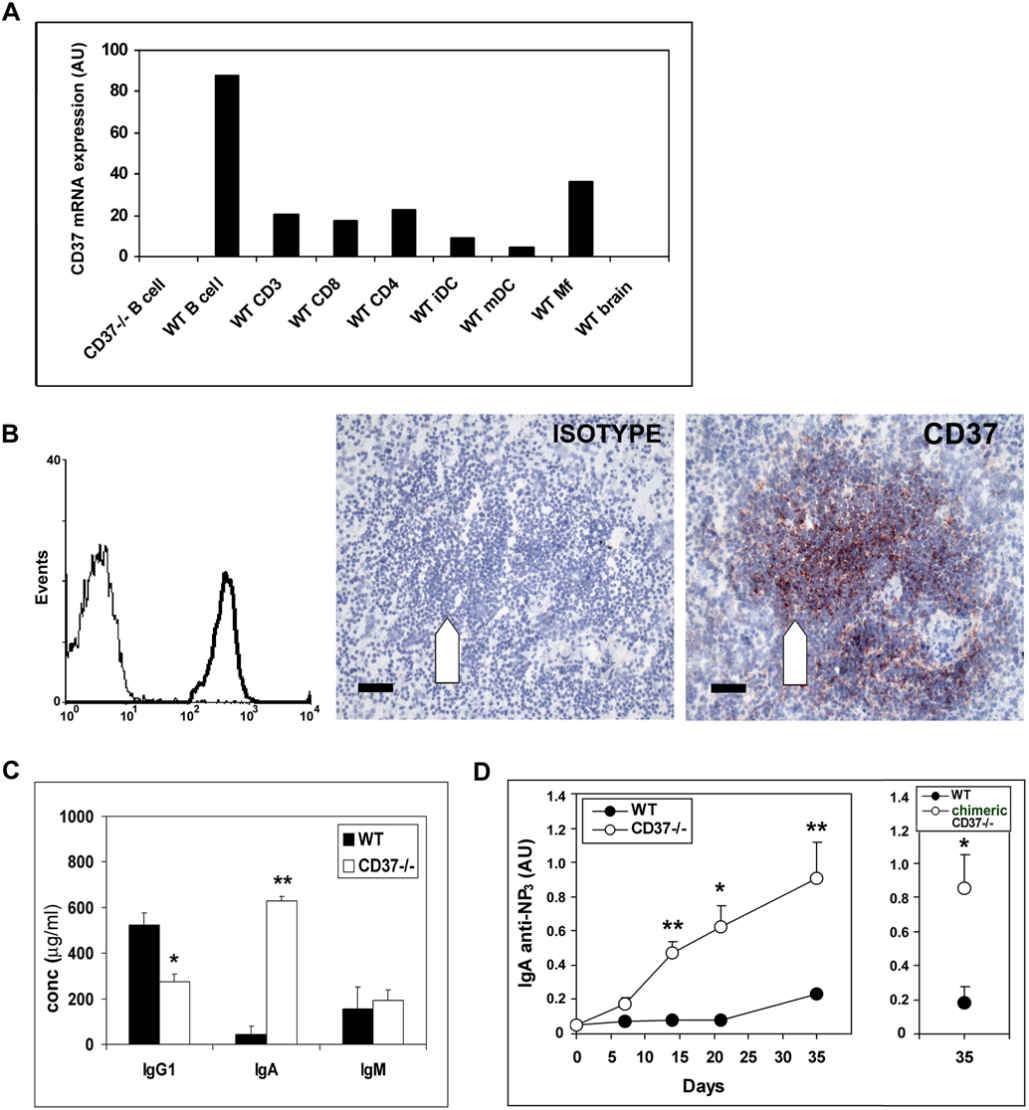
Increased IgA production in CD37-deficient mice. (A) Murine CD37 mRNA expression (arbitrary units) on various MACS-purified leukocyte subsets determined by quantitative RT–PCR. i/mDC = (im)mature dendritic cell, mf = macrophage. (B) Human CD37 expression on peripheral blood CD19^+^ B cells determined by flow cytometry (left) and in B cell follicle area (arrow) of spleen determined by immunohistochemistry (right). Scale bar is 50 µm. Similar CD37 expression was found in lymph nodes. (C) Sera of non-immunized WT (black) and CD37−/− (white) mice were analyzed for the amount of immunoglobulin isotypes by ELISA. Antibody titer is expressed in arbitrary units and represented as mean±SEM (n = 9). Asterisks indicate significant difference (*p<0.0002, **p<0.00005). (D) WT and CD37−/− mice were immunized with NP-KLH and sera were assayed for high affinity NP-specific IgA by ELISA (left). IgA response in chimeric mice that contain either WT or CD37-deficient B cell compartment 35 d after immunization (right). Antibody titer is expressed in arbitrary units and represented as mean±SEM (n = 6). Asterisks indicate significant difference as per: *p<0.03 and **p<0.001. Similar results were obtained for total (low and high affinity) NP-specific IgA.

CD37 expression was determined in different mouse and human leukocyte populations at the mRNA and protein level, respectively. In both mice and human, B cells were found to be the major CD37 expressing cells (Figure 1A and 1B). In lymphoid tissue, CD37 expression was highest in the B cell follicle area as expected (Figure 1B, data shown for spleen). This high CD37 expression on B cells prompted us to study humoral immunity in CD37-deficient mice in detail. When analyzing the basal antibody levels in sera of naïve mice, we observed that basal levels of serum IgA in CD37−/− mice were increased more than 15-fold compared to C57BL/6J wild-type (WT) mice (Figure 1C). At the same time, IgG1 levels were decreased 2-fold and serum IgM levels were unaltered compared to WT mice. Next, we investigated antibody responses after immunization with the T cell-dependent antigen (NP-KLH) in CD37−/− and WT mice. Sera were analyzed for the presence of antibodies reactive with NP_3_-BSA (high affinity anti-NP antibody) and NP_20_-BSA (total anti-NP antibody) [20]. Again, CD37−/− mice developed high titers of anti-NP IgA, with >10-fold increase of high affinity IgA compared to controls 21 days after immunization (Figure 1D).

### B cells lacking CD37 are responsible for the high IgA production

We wanted to investigate whether CD37-deficiency on the B cell population was indeed responsible for the high IgA response. WT mice were sublethally irradiated and reconstituted with bone marrow; 80% from μMT mice (B cell-deficient mice), and 20% from WT or CD37−/− mice as a source of B cells. This created WT mice and mice with a CD37-deficient B cell compartment (referred to as chimeric CD37−/− mice). The percentages of CD19-, CD3-, GR-1-, and F4/80-expressing cells assessed in peripheral blood revealed equally efficient reconstitution in WT and chimeric CD37−/− mice (data not shown). Next, NP-specific antibody production was analyzed 35 days after immunization with NP-KLH. The results clearly demonstrate that chimeric mice with a CD37−/− B cell repertoire also produced high titers of NP-specific IgA comparable to intact CD37−/− mice after immunization (Figure 1D, right). Thus, we can conclude that the elevated IgA response in CD37−/− mice is a B cell-intrinsic defect.

### Increased formation of IgA–secreting plasma cells in CD37−/− mice

**Figure 2:**
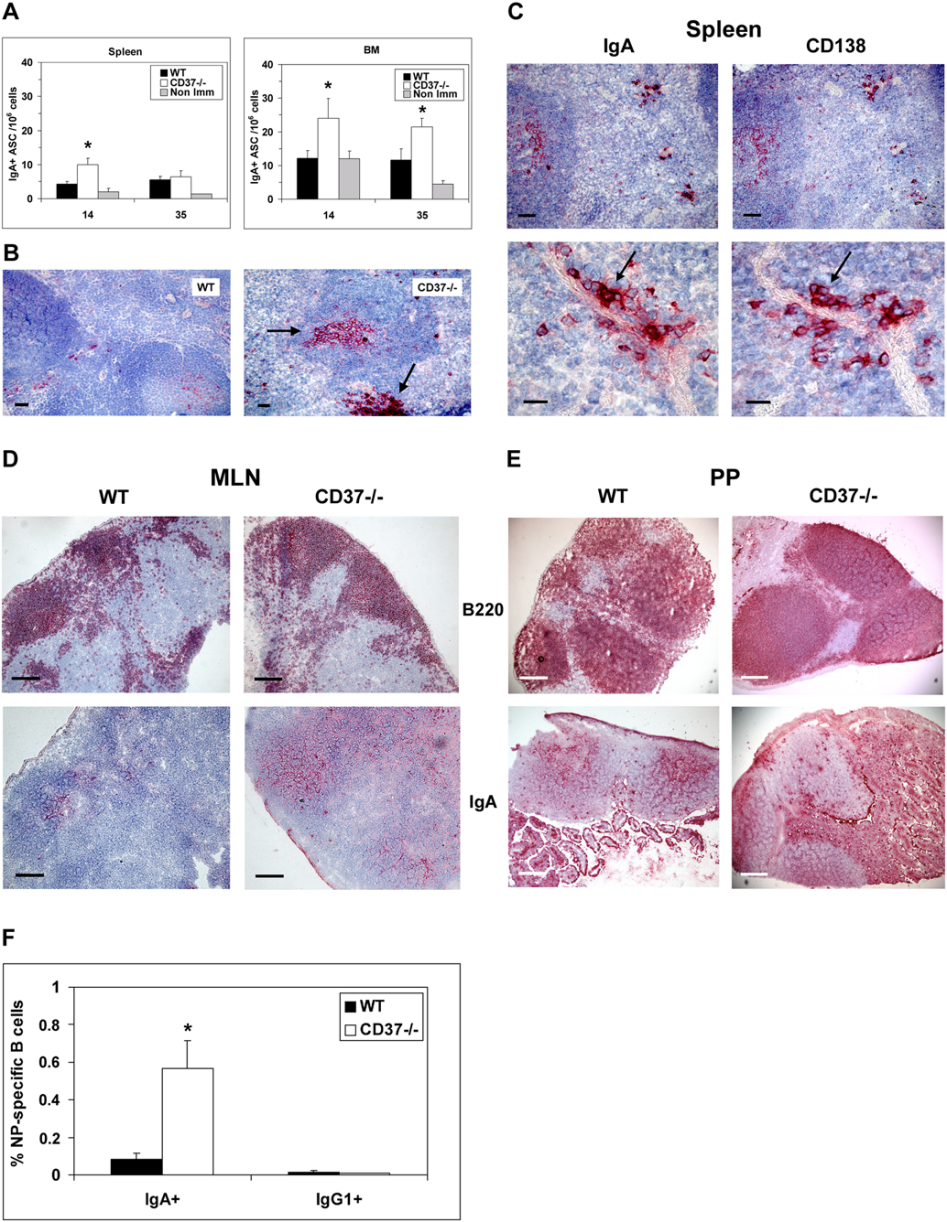
Increased formation of IgA–secreting plasma cells in CD37−/− mice. (A) The frequency of high affinity NP-specific IgA–ASC was assessed in CD37−/− (white) and WT (black) spleen and bone marrow by ELISPOT at d 14 and 35 of the immune response. Non-immunized mice are shown as control (grey). Data are presented as mean±SEM (n = 6). Asterisks indicate significant differences at p<0.05. (B) Immunohistochemical analysis of WT (left) and CD37−/− spleens (right) demonstrating IgA–positive plasma cells (arrows) at d 14 after immunization. Scale bar is 50 µm. (C) Immunohistochemical analysis of serial sections from spleens of CD37−/− mice stained with antibodies specific for IgA (left) and plasma cell marker CD138 (right) at d 14 after immunization. Arrows indicate co-localization. Scale bar is 50 µm (upper) and 15 µm (lower). Mesenteric lymph nodes (D) and Peyer's patches (E) of WT and CD37−/− mice were analyzed by immunohistochemistry at 14 d after NP-KLH immunization using antibodies specific for B cells (upper) and IgA (lower). Note the increased numbers of IgA–positive cells in CD37−/− tissue. Scale bars are 300 µm. (F) Percentage of IgA^+^ and IgG1^+^ NP-specific plasma cells in Peyer's patches of WT (black) and CD37−/− (white) mice determined by flow cytometry at d 21 after immunization. Data are presented as mean±SEM (n = 4). Asterisk indicates statistical difference at p<0.05.

The high IgA titers in serum of CD37−/− mice suggest an enhanced generation of IgA-secreting plasma cells. To determine if this was the case, spleens, bone marrow, and mucosal-associated lymphoid tissue from immunized mice were examined for the frequency of NP-specific IgA-ASC using ELISPOT assays. In line with IgA levels in serum, the numbers of IgA-ASC were increased in spleen of CD37−/− mice compared to control WT mice at day 14 following immunization (Figure 2A). This observation was confirmed by immunohistochemical analysis (Figure 2B). High numbers of IgA^+^ cells were present in GC and white pulp area in spleens of immunized CD37−/− mice, whereas in spleens of WT mice IgA^+^ cells were very rarely detected. CD138 stainings confirmed that the IgA^+^ cells in CD37−/− spleens were indeed plasma cells (Figure 2C). Since IgA-ASC are known to migrate preferentially to the MALT using specific gut-homing receptors [21], Peyer's patches and mesenteric lymph nodes of immunized CD37−/− mice were analyzed for the presence of NP-specific IgA-ASC. Both the morphology and organization of mesenteric lymph nodes (Figure 2D) and Peyer's patches (Figure 2E) of CD37−/− mice were comparable to WT mice, although CD37−/− Peyer's patches were slightly increased in size. The percentage of IgA^+^ NP-specific plasma cells was increased in MALT of immunized CD37−/− mice compared to WT controls (Figure 2F), demonstrating that IgA-ASC generated in CD37−/− spleens preferentially homed to the MALT rather than to the bone marrow. IgG1^+^ NP-specific plasma cells were hardly detected in MALT of CD37−/− and WT mice as expected. Taken together, CD37−/− mice possess increased generation of antigen-specific IgA^+^ plasma cells after immunization.

### Elevated IgA responses in CD37−/− mice are dependent on IL-6

**Figure 3:**
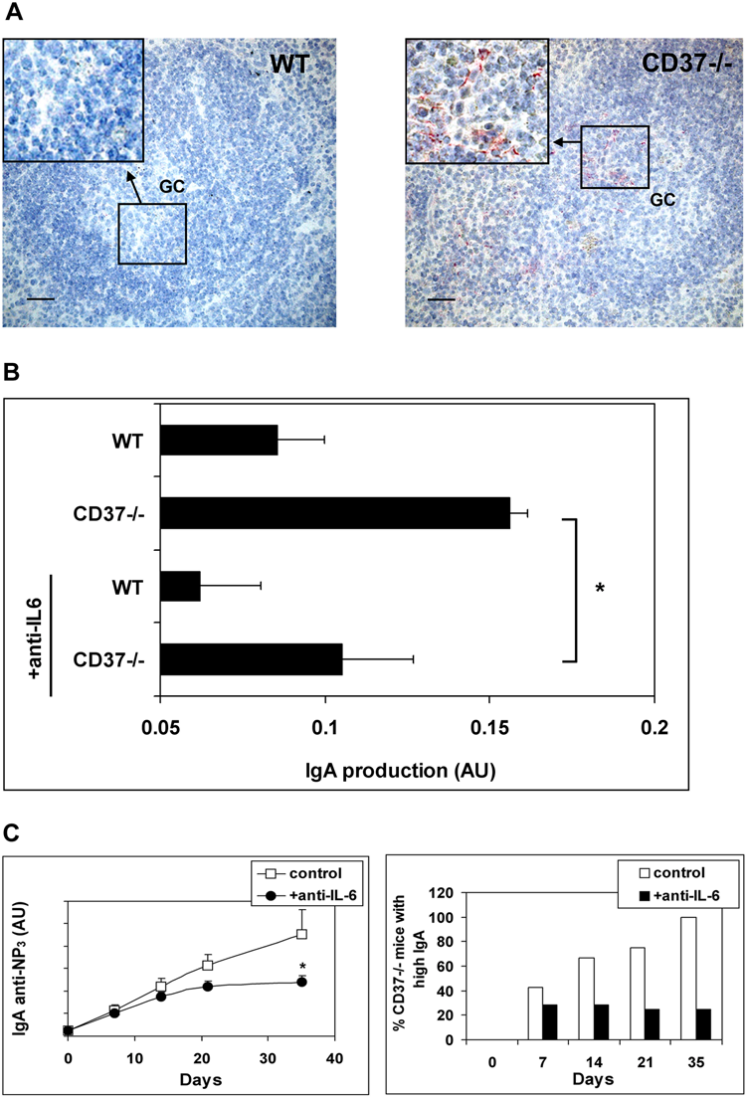
Increased IgA production after immunization in CD37−/− mice is dependent on IL-6. (A) Immunohistochemical analysis of spleens from CD37−/− and WT mice 14 d after NP-KLH immunization. Germinal centers (identified in serial sections by PNA staining) are indicated in the B cell follicles. Immunized WT mice have non-detectable levels of IL-6 in spleens (left). In contrast, B cell follicles in spleens of immunized CD37−/− mice are clearly positive for IL-6 (red) in the GC area (right). Spleens of non-immunized mice (WT and CD37−/−) were negative for IL-6 staining (not shown). Scale bar is 50 µm. (B) Effect of neutralizing anti–IL-6 during *ex vivo* restimulation. Splenocytes from WT and CD37−/− mice were prepared 14 d after NP-KLH immunization, and stimulated *in vitro* with NP-KLH (1 µg/ml) in the absence or presence of anti–IL-6. Supernatants were collected after 48 h, and assayed for IgA production by ELISA (expressed in arbitrary units). Asterisk indicates significant difference (*p<0.002). (C) IL-6 was neutralized in WT and CD37−/− mice during immunizations using blocking IL-6 antibodies (as described in Materials and methods). High affinity NP-specific IgA was assayed in serum of CD37−/− mice treated with anti–IL-6 (black) or control antibody (white) (left). Antibody titer is expressed in arbitrary units and represented as mean±SEM (n = 6). Asterisks indicate significant difference as per: *p<0.04. Histogram shows percentage of CD37−/− mice with high IgA anti-NP_3_ levels (above 10× background level) in serum after treatment with anti–IL-6 (black) compared to control treated CD37−/− mice (white) at indicated days after immunization (right). Similar results were obtained for total NP-specific antibody (against NP_20_-BSA).

IL-6 is a cytokine that has been implicated in promoting the generation of IgA-secreting plasma cells [2],[3]. Moreover, we have recently shown that signals transduced through the CD37 molecular partner, dectin-1, lead to an elevated production of IL-6 by CD37−/− cells [17]. Consequently, we examined whether a dysregulation of IL-6 production in immunized CD37−/− mice may underlie the excess production of IgA. We first examined the expression of IL-6 in the germinal centers (GC) of immunized mice by immunohistochemistry. We readily detected IL-6 expression in the GC area of spleens of immunized CD37−/− (Figure 3A). In contrast, non-immunized mice (WT and CD37−/− mice) as well as immunized WT mice had non-detectable levels of IL-6 in spleens. IgA production by peritoneal B1 cells during T cell-independent responses is not dependent on IL-6 [22], which correlates with the normal IgA response in CD37−/− mice after T cell-independent immunization (data not shown).

Next, the effect of IL-6 on IgA production during *ex vivo* restimulation experiments was analyzed. Splenocytes of immunized WT and CD37−/− mice were stimulated with NP-KLH *in vitro* in the absence or presence of neutralizing IL-6 antibodies. Figure 3B shows increased IgA production by CD37−/− cultures compared to WT cells as expected. Blocking IL-6 resulted in substantially reduced IgA production by CD37−/− cells, which supported our hypothesis that the mechanism underlying the elevated IgA responses in CD37−/− mice is controlled at the level of IL-6. WT and CD37−/− cultures produced 1900 vs. 5500 pg/ml IgA respectively, which decreased to 500 vs. 2000 pg/ml in the presence of neutralizing IL-6 antibodies. We also established that purified CD37−/− splenic B cells were capable of autocrine IL-6 production upon restimulation *in vitro* using intracellular cytokine stainings (data not shown).

To prove that increased IgA production in CD37−/− mice was indeed dependent on IL-6 *in vivo*, we neutralized IL-6 in WT and CD37−/− mice during immunizations using blocking antibodies. Serum was analyzed for the presence of high affinity (anti-NP_3_) IgA antibodies at different days after immunization. Evidently, IL-6 neutralization reversed the production of high IgA levels in CD37−/− serum (Figure 3C, left), demonstrating the importance of IL-6 in IgA^+^ plasma cell development in CD37−/− mice. Only 25% of the CD37−/− mice produced NP-specific IgA titers after treatment with anti–IL-6 antibodies, compared to 100% of isotype control-treated CD37−/− mice 14 days after immunization (Figure 3C, right). IgA formation by WT mice (with or without anti–IL-6) was below the level of detection (not shown). Our findings are in accordance with observations made in IL-6-deficient mice that exhibit impaired antibody production during systemic immune responses [23]–[25]. Mucosal IgA responses were reported to be significantly impaired or unaltered in IL-6-deficient mice dependent on the model of immunization used [2],[23],[25],[26]. Moreover, IL-6 polymorphisms were recently reported to be associated with IgA-deficiency in patients [27]. Since IL-6 is crucial for the induction of already-committed (surface IgA^+^) B cells to become IgA-secreting plasma cells [2]), our data suggest that CD37 is directly implicated in the inhibition of the late-stage development of IgA^+^ plasma cells, i.e. at the level of terminal B cell differentiation. Taken together, we established that high IL-6 levels directly relate to the increased IgA production in CD37−/− mice.

### IgA production during fungal infection in CD37-deficient mice

Given that IgA contributes to protecting the host from microbial infections, we hypothesized that CD37-deficiency may have important implications for the outcome of infectious diseases. We have recently demonstrated that CD37 interacts with the C-type lectin dectin-1 [17], a β-glucan receptor that is required for effective immunity to fungal infections [28],[29]. Therefore, the CD37-deficient immune response during *Candida albicans* infection was explored. *C. albicans* normally colonizes the mucosa without causing disease, but can cause systemic infection with high mortality in immunocompromised patients [30],[31]. In particular, the incidence of invasive *C. albicans* infections is high among cancer patients [32]–[34].

**Figure 4:**
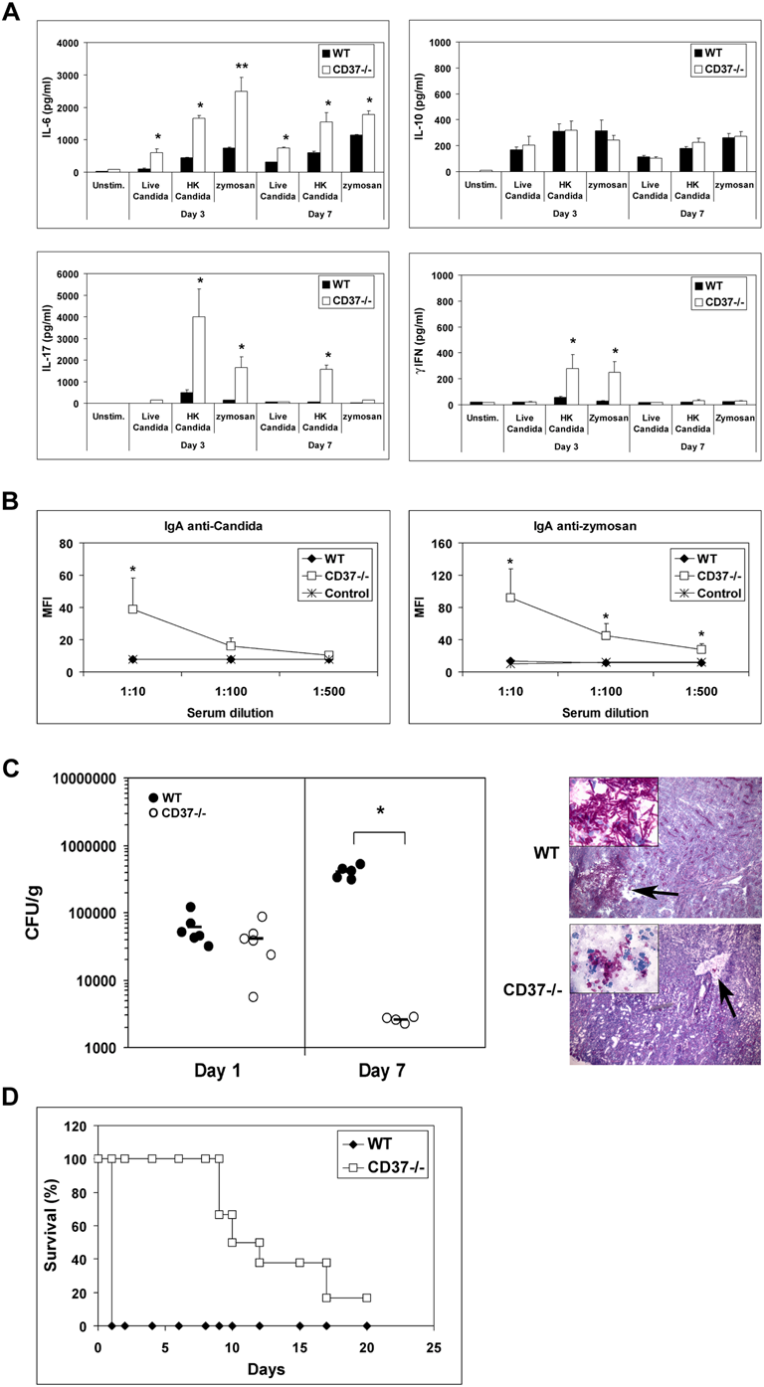
CD37−/− mice are protected against *C. albicans* infection. CD37−/− and WT mice (n = 5) were systemically infected with 1×10^5^ *C. albicans*. (A) Spleens were removed 3 or 7 d after infection and splenocytes were stimulated with live, heat-killed *C. albicans* yeasts, zymosan (E∶T ratio 2∶1) for 48 h, after which IL-6, IL-10, IL-17, and γIFN in supernatants were measured by ELISA. Asterisks indicate significant difference as per: *p<0.05 and **p<0.002. (B) Serum of CD37−/− and WT mice was analyzed for the presence of IgA antibodies reactive with *C. albicans* yeast (left) or zymosan (right) by flow cytometry as described in Materials and methods. Serum of non-infected mice is shown as control. Asterisks indicate significant difference as per: *p<0.05. (C) left: Subgroups of five or six animals were sacrificed on d 1 or 7, and kidneys were analyzed for the number of viable *Candida* cells (expressed as log CFUg^−1^ tissue). Horizontal bars represent mean. Asterisks indicate significant difference as per: *p<0.0005. Right: Histology of kidneys from WT and CD37−/− mice 7 d after infection. Note the major infection area and infiltrating hyphae in WT kidneys. (D) Survival of WT and CD37−/− mice (n = 6) was assessed after intravenous injection of lethal dose of 5×10^5^ CFU *C. albicans*.

CD37−/− and WT mice were infected with *C. albicans* and IL-6 production by CD37−/− and WT splenocytes was studied upon restimulation with fungal antigens. CD37−/− splenocytes produced increased levels of IL-6 compared to WT cells upon exposure to either live or heat-killed *C. albicans*, or the fungal cell wall extract zymosan both 3 and 7 days after infection (Figure 4A). Blocking dectin-1 with antibody 2A11 inhibited IL-6 production by both WT and CD37−/− splenocytes stimulated with *C. albicans* or the dectin-1 ligand curdlan (Figure S1), showing that IL-6 production is dependent on dectin-1. As such, CD37 controls dectin-1-mediated IL-6 production, possibly by recruiting dectin-1 into tetraspanin microdomains that may alter signal transduction pathways and subsequent cytokine profiles. In line with our findings, IL-6-deficient mice are more susceptible to *C. albicans* and *A. fumigatus* infection, which is related to decreased neutrophil effector activity, impaired Th1-mediated immune responses [25], and defective Th17 responses [35]. Studying Th2/Th1/Th17 cytokine production by CD37−/− splenocytes revealed that IL-10 production was comparable between CD37−/− and WT splenocytes, and γIFN production was low but increased by CD37−/− cells 3 days after infection (Figure 4A). The role of IL-6 in inducing Th17 responses is well established in mice. Accordingly, we observed significantly increased IL-17 production by CD37−/− splenocytes upon *C. albicans* stimulation (Figure 4A). Th17 cells have been implicated as an important effector mechanism against *C. albicans* infection [36],[37], although IL-17 may also impair anti-fungal immunity under certain conditions [38],[39].

Next, the development of fungal-specific IgA antibodies in CD37−/− and WT mice exposed to *C. albicans* was analyzed. WT mice did not generate a detectable IgA response after *C. albicans* after infection. In contrast, all CD37−/− mice exhibited high titers of IgA antibodies specific for *C. albicans* and zymosan in their serum (Figure 4B). *Candida*-specific IgG was not detected in serum of WT and CD37−/− mice 7 days after infection (not shown). Finally, we investigated the role of tetraspanin CD37 in the outcome of infectious disease. WT and CD37−/− mice were systemically infected with *C. albicans* yeasts, and kidneys were analyzed for the outgrowth of viable *C. albicans* after 1 and 7 days. CD37−/− mice exhibited significantly decreased susceptibility to infection and reduced fungal outgrowth in their kidneys –the main target organ for *C. albicans* - 7 days after infection when compared to WT mice (Figure 4C, left). Histology revealed major infection areas with abscesses and hyphal infiltration in WT kidneys. In contrast, morphology of kidneys of CD37−/− mice looked normal with no mycelial structures and presence of leukocyte infiltrates (Figure 4C, right). Next, we evaluated survival of WT and CD37−/− mice after lethal fungal infection. The absence of CD37 resulted in prolonged survival and decreased mortality after lethal candidiasis (Figure 4D), further emphasizing the importance of CD37 in regulating anti-fungal immunity.

**Figure 5:**
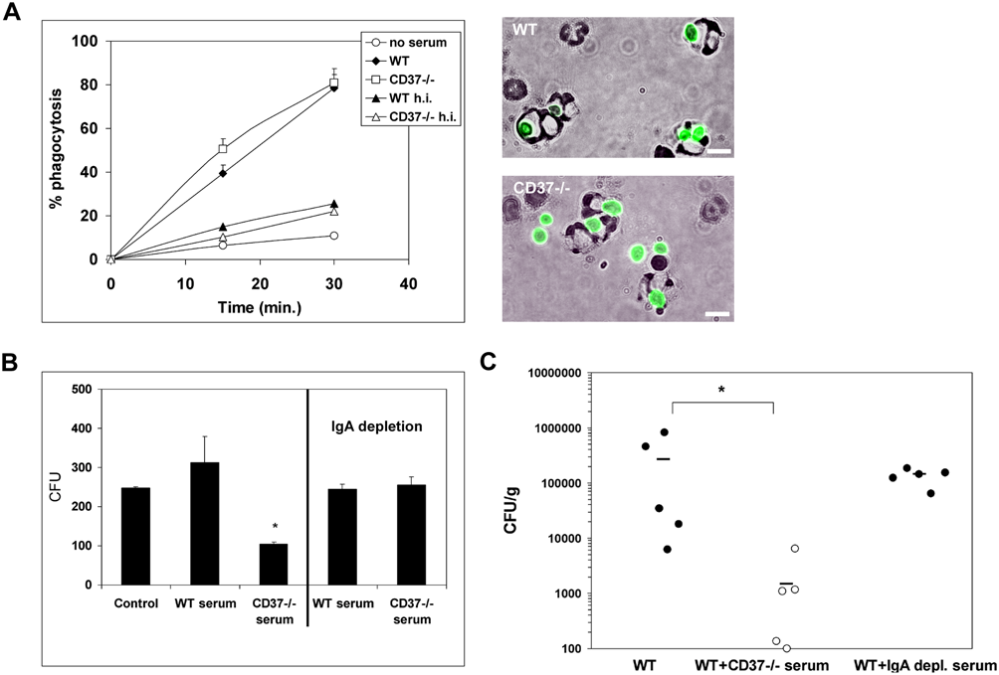
Adoptive transfer of CD37−/− serum increases resistance of WT mice to fungal infection. (A) Percentage of GR-1^+^ blood leukocytes that phagocytosed FITC-labeled *C. albicans* in the absence or presence of serum from infected WT or CD37−/− mice for various time points at 37°C was quantified by flow cytometry. Sera were heat-inactivated (56°C for 30 min) to inactivate complement (left). In addition, uptake in the presence of WT or CD37−/− serum was analyzed by fluorescence light microscopy (right; data shown for 30-min. incubation). Bar represents 10 µm. (B) *C. albicans* was incubated in the absence or presence of 20% serum (from infected WT or CD37−/− mice) or 20% IgA–depleted serum and growth (CFU) was assessed. Asterisks indicate significant difference as per: p<0.005. (C) WT mice (n = 5) were systemically infected with 1×10^5^ *C. albicans* and untreated, or treated with either pooled CD37−/− serum containing high levels of fungal-specific IgA, or IgA-depleted CD37−/− serum. Kidneys were analyzed for the number of viable *Candida* cells (expressed as log CFUg^−1^ tissue) 7 d after infection. Horizontal bars represents mean. Asterisks indicate significant difference as per: p<0.05.

In order to get more insight into the underlying mechanism, CD37−/− serum was analyzed for fungal opsonization and/or neutralizing capacity. IgA-mediated phagocytosis through Fc alpha receptor CD89 is well-established in humans [40],[41], in whom serum IgA is mostly monomeric. In mice, serum IgA is predominantly dimeric/polymeric and it is currently unknown whether the murine Fcα/μ receptor is effective in clearing IgA-opsonized pathogens [42]. We observed similar uptake of *C. albicans* by GR-1^+^ neutrophils in the presence of WT or CD37−/− serum of infected mice (Figure 5A). Serum heat-inactivation inhibited *C. albicans* uptake, demonstrating that the phagocytosis was mainly complement-mediated and not dependent on IgA. Also, the increase in GR-1-expressing cells in blood after *C. albicans* infection was comparable between WT and CD37−/− mice (Figure S2). Thus, these data demonstrate that IgA in CD37−/− serum does not increase *C. albicans* phagocytosis by neutrophils.

Next, we investigated whether serum IgA of CD37−/− mice could directly neutralize fungal cells. *C. albicans* was grown in the absence or presence of WT and CD37−/− serum of infected mice and viability was assessed by classical plate assays. CD37−/− serum significantly inhibited *C. albicans* growth in contrast to WT serum (Figure 5B). To demonstrate that the growth inhibition was due to fungal-specific IgA, CD37−/− serum was depleted for IgA. Serum deprived from IgA abrogated the effect, and *C. albicans* growth was comparable between WT and CD37−/− serum. From these findings we conclude that IgA in CD37−/− serum directly neutralizes *C. albicans* and may thereby prevent dissemination *in vivo*.

To test this hypothesis, WT mice were infected and treated with pooled CD37−/− serum containing high levels of IgA anti-*C. albicans*. We observed that WT mice were protected from fungal disease by CD37−/− serum treatment (Figure 5C). Importantly, the resistance was abrogated when IgA was depleted from CD37−/− serum, demonstrating that the passive transfer of resistance to candidiasis in CD37−/− mice is dependent on IgA. Although the elevated IL-6-IL-17 response may also contribute to increased anti-fungal effector activity in CD37−/− mice (Figure 4A), our passive transfer data clearly show that *C. albicans*-specific IgA alone is sufficient to confer protection against invasive fungal disease. Whilst several studies have alluded to a role for the development of protective anti-fungal antibodies in experimental candidiasis [43],[44], this is—to the best of our knowledge—the first description of an important role for IgA in protection against systemic candidiasis. These studies also highlight the non-redundant role of the tetraspanin CD37, as CD37-deficiency stimulates anti-fungal immunity even in the presence of other tetraspanins. The results of this study may stimulate the development of novel immunotherapeutic strategies for invasive fungal disease that are urgently warranted [33].

In summary, we reveal a unique role for the tetraspanin CD37 in the humoral immune response; CD37 is a negative regulator of IgA^+^ plasma cell generation *in vivo* and this role is critically dependent on IL-6. Moreover, this is the first demonstration that tetraspanins may control the immune-mediated defense against fungal pathogens. This study increases our understanding of molecular mechanisms underlying the formation of IgA-secreting plasma cells and may contribute to better insight into anti-fungal immunity.

## Materials and Methods

### Quantitative RT–PCR

Murine CD37 expression was analyzed at the mRNA level by quantitative RT–PCR due to unavailability of anti-mouse CD37 antibodies. Total RNA was extracted from WT and CD37−/− leukocyte populations (purified by MACS sorting, Miltenyi Biotec) using Trizol (Invitrogen) and transcribed into cDNA using random hexamer primers (Amersham) and Superscript II RT (Invitrogen). Brain tissue was taken as negative control. Real-time RT–PCR was performed in ABI Prism Sequence Detection system 7000. cDNA was amplified using SYBR Green PCR mastermix (Applied Biosystems) with mouse CD37 primers (5′GTCCTTTGTGGGTTTGTCCTT and 5′GAGACAGCGCAGCTCCTTTAG). The amount of CD37 mRNA expression was normalized to the housekeeping gene PBGD.

### Human CD37 expression

Peripheral blood lymphocytes were stained with anti-CD37 (clone WR17, Serotec) and processed for FACS analysis as previously described [17]. CD37 staining in tissue was performed on frozen sections (6 µm) of spleen and lymph node that were fixed in acetone for 10 minutes at −20°C. Slides were blocked (5% horse serum) and stained with anti-CD37 or mIgG2a isotype control. Horse anti-mouse-biotin (Molecular Probes) was used as secondary Ab, and staining was revealed using SA-alkaline phosphatase labeling kit (Vector Laboratories) with Fast Red substrate. Slides were counterstained with Meyer's heamatoxylin.

### Mice

CD37−/− mice were generated by homologous recombination [14] and backcrossed 10 times to the C57BL/6J background. Age and sex-matched C57BL/6J wild type (WT) mice were obtained from the Walter and Eliza Hall Institute (Melbourne, Victoria, Australia) and Charles River (France). CD37−/− mice were bred at the Burnet Institute Austin Campus (Heidelberg, Victoria, Australia) and the Central Animal Laboratory (Radboud University Nijmegen, The Netherlands). μMT (B cell-deficient) mice [45] were bred at the Walter and Eliza Hall Institute. Mice were used at 8–12 weeks of age. Animal studies were approved by animal ethics Committee of the Austin&Repatriation Medical Centre, and the Nijmegen Animal Experiments Committee.

### Immunization

WT and CD37−/− mice (n = 6) were injected intraperitoneally with 100 µg of NP (4-hydroxy-3-nitrophenylacetate) coupled to Keyhole Limpet Hemocyanin (KLH) (NP_17_∶KLH, conjugation ratio, 17∶1) precipitated in alum. Mice were bled and sera collected at days 7, 14, 21 and 35 post-immunization. Lymphoid organs (spleens, bone marrow, mesenteric lymph nodes, and Peyer's patches) were frozen for immunohistochemistry or processed for FACS analysis.

### Detection of Ag-specific immunoglobulin and antibody-secreting cells

Basal immunoglobulin levels in serum of non-immunized mice (n = 9) were determined by ELISA using goat anti-mouse IgG/IgA/IgM (BD-Pharmingen). Ig standards were purchased from BD. Levels of NP-specific antibodies in serum after immunization were assayed by ELISA using plates coated with 20 µg/mL of either NP_20_-BSA or NP_3_-BSA to detect total and high affinity anti-NP antibody, respectively. The frequency of total and high affinity NP-specific ASC in spleen and bone marrow was determined by ELISPOT as described [20],[46], again using NP_20_-BSA and NP_3_-BSA for antibody capture.

### Immunohistochemistry

Spleens, mesenteric lymph nodes and Peyer's patches taken from mice before and after immunization were embedded in OCT. Frozen sections (6 µm) were fixed in acetone for 10 min. at −20 C. Slides were blocked (5% goat serum) and stained with anti-IgA, anti-IgG1, anti-IL6, anti-CD138, anti-CD45R (B220) (all from BD-Pharmingen), or isotype controls. Goat anti-rat-biotin (Molecular Probes) was used as secondary Ab, and staining was revealed using SA-alkaline phosphatase labeling kit (Vector Laboratories) with Fast Red substrate. Slides were counterstained with Meyer's heamatoxylin.

### Reconstitution experiments

Bone marrow chimeras were generated as previously described [47]. Briefly, 10^7^ (CD37−/− or WT) and μMT (CD37+) bone marrow cells were injected intravenously into sublethally irradiated C57Bl/6J recipients at a ratio of 4 parts μMT to 1 part WT or CD37−/− bone marrow as a source for B cells. This created WT mice and mice with a CD37-deficient B cell compartment (referred to as chimeric CD37−/− mice). Six weeks after reconstitution, heparinized blood was obtained from chimeric mice and reconstitution of all major leukocyte populations was monitored by flow cytometry, using the antibodies against: PE-conjugated 1D3-PE (CD19, BD-Pharmingen), PE-conjugated RB6-8C5-PE (GR-1, BD-Pharmingen), Cy5-conjugated KT3.1 (CD3, made in house), PE-conjugated F4/80 (BD-Pharmingen), and FITC-conjugated M1/70 (CD11b, made in house). 2 weeks thereafter, mice (n = 7) were immunized with NP-KLH as described above. Sera were collected 35 days after immunization and analyzed for presence of NP-specific antibodies by ELISA.

### *Ex vivo* restimulation experiments

Splenocytes were prepared from WT and CD37−/− mice 14 days after NP-KLH immunization. Cells were stimulated with NP-KLH (1 µg/ml) in the absence or presence of 5 µg/ml (azide- and LPS-free) neutralizing anti–IL-6 MP5-20F3 (Biolegend). Supernatants were collected after 48 h, and IgA production was determined by ELISA.

### IL-6 neutralization *in vivo*

WT and CD37−/− mice (n = 6) were immunized with NP-KLH as described above. Mice received 2 injections of neutralizing (azide- and LPS-free) anti–IL-6 MP5-20F3 or isotype control; 60 µg intraperitoneally at day 0, and 60 µg intravenously 7 days after immunization. Mice were bled and sera collected at days 7, 14, 21 and 35 post-immunization. Lymphoid organs (spleens, bone marrow, mesenteric lymph nodes, and Peyer's patches) were frozen in OCT for immunohistochemistry or processed for FACS analysis.

### *Candida albicans* infection protocols

*C. albicans* ATCC MYA-3573 (UC820), a well-described clinical strain [48], was injected intravenously (1×10^5^ colony-forming units (CFU), in 100 µL sterile pyrogen-free saline) in CD37−/− and WT mice. Blood (50 µl) was collected at different time points after infection and stained with GR-1-PE to identify neutrophils by flow cytometry. Subgroups of five or six animals were sacrificed on day 1 or 7, and kidneys were removed aseptically, weighed and homogenized in sterile saline in a tissue grinder. The number of viable *Candida* cells in the tissues was determined by plating serial dilutions on Sabouraud dextrose agar plates. Colony-forming units were counted after 24 h of incubation at 37°C, and expressed as log CFUg^−1^ tissue. Alternatively, kidneys were were embedded in OCT, and frozen sections (6 µm) were stained with periodic acid-Schiff to identify *C. albicans* by histology. In adoptive transfer experiments, WT mice (n = 5) received 1×10^5^ CFU *C. albicans* intravenously (in 100 µL sterile pyrogen-free saline) with 20% pooled serum of infected CD37−/− mice that was either untreated or depleted for IgA (as described below). Colony-forming units were determined in kidneys 7 days after infection. For survival experiments, CD37−/− and WT mice (n = 6) were injected intravenously with lethal dose of 5×10^5^ CFU *C. albicans* and mice were assessed twice daily.

### Restimulation assay with fungal antigens

To assess cytokine production, spleens were removed 3 or 7 days after infection and 5×10^6^ splenocytes were stimulated with 1×10^7^ live or heat-killed *C. albicans* yeasts (E∶T ratio 2∶1), or 1 mg/ml zymosan (Sigma). Measurement of IL-6, IL-10, IL-17 and γIFN was performed in supernatants collected after 48 h of incubation at 37°C (5% CO_2_) in 24-well plates by commercial ELISA assays (Biosource, Camarillo, CA; detection limit 16 pg/ml), according to the instructions of the manufacturer. In dectin-1 blocking studies, antibody 2A11 (10 µg/ml) was added during stimulations as described [17]. Curdlan is a specific dectin-1 ligand with no TLR2- or TLR4-stimulating properties [49].

### Detection of anti-*Candida* IgA antibodies in mice

Serum of CD37−/− and WT mice (n = 5) was collected 7 days after *C. albicans* infection, and different dilutions were incubated with 1×10^5^ *Candida* or 1×10^5^ zymosan particles for 30 min. at 4°C. After washing twice in saline, *Candida* particles were incubated with biotinylated anti-mouse IgA (BD-Pharmingen) followed by SA-CY5 (Invitrogen), and analyzed by flow cytometry. Serum of non-infected mice was used as control.

### IgA depletion of serum

Streptavidin-coated 2 µm microspheres (Polysciences, PA) were incubated with biotinylated anti-mouse IgA (BD Biosciences) according to the instructions of the manufacturer. Sera of WT and CD37−/− mice (n = 4) were collected 7 days after *C. albicans* infection, pooled, and incubated with 100×10^6^ anti-IgA coated microspheres (1 h at RT, shaking).

### *C. albicans* phagocytosis and neutralization assays

*C. albicans* phagocytosis was performed as described [41]. Briefly, FITC-labeled *C. albicans* particles were incubated with blood leukocytes (ratio 4∶1) in the absence or presence of serum from infected WT or CD37−/− mice for various time points at 37°C. As control, sera were heat-inactivated at 56°C for 30 min. to inactivate complement. GR-1-PE was used to stain neutrophils and phagocytosis was quantified by flow cytometry. In addition, uptake was analyzed by fluorescence light microscopy. For neutralization experiments, *C. albicans* was cultured overnight at 37°C in Sabouraud dextrose medium (Oxoid, UK), washed, and 1×10^5^ yeasts were incubated in the absence or presence of 20% serum (from WT or CD37−/− mice; collected 7 days after infection) or 20% IgA-depleted serum. Viability was assessed after shaking for 5 h at 37°C by plating serial dilutions on Sabouraud dextrose agar plates.

### Statistical analyses

Data are presented as mean±SEM. Statistical differences were determined using the unpaired Student's t-test. Significance was accepted at the p<0.05 level.

### Accession/ID numbers

Human CD37, GeneID 951; Murine CD37, GeneID 12493, MGI: 88330; Murine IL-6, GeneID: 16193, MGI: 96559; Murine IgA, GeneID: 238447, MGI: 96444.

## Supporting Information

Figure S1 IL-6 production by WT and CD37−/− splenocytes stimulated by *C. albicans* is dependent on dectin-1. Spleens were removed from CD37−/− and WT mice 7 d after infection (1×10^5^ CFU *C. albicans*) and restimulated with heat-killed *C. albicans* (E∶T ratio 2∶1) or the dectin-1 ligand Curdlan (100 µg/ml) for 48 h, after which IL-6 in supernatants were measured by ELISA. Antibody 2A11 was added during stimulations (10 µg/ml) to block dectin-1.
(0.01 MB PDF)

Figure S2 Normal increase of GR-1-positive cells in blood of CD37−/− mice. Granulopoiesis in WT and CD37−/− mice (n = 5) systemically infected with 1×10^5^ CFU *C. albicans*. Percentage of GR-1-positive cells in blood was determined by flow cytometry at different time points after infection.
(0.01 MB PDF)

## References

[1] Hirano T, Taga T, Nakano N, Yasukawa K, Kashiwamura S (1985). Purification to homogeneity and characterization of human B-cell differentiation factor (BCDF or BSFp-2).. Proc Natl Acad Sci U S A.

[2] Ramsay AJ, Husband AJ, Ramshaw IA, Bao S, Matthaei KI (1994). The role of interleukin-6 in mucosal IgA antibody responses in vivo.. Science.

[3] Mora JR, Iwata M, Eksteen B, Song SY, Junt T (2006). Generation of gut-homing IgA-secreting B cells by intestinal dendritic cells.. Science.

[4] McGhee JR, Kunisawa J, Kiyono H (2007). Gut lymphocyte migration: we are halfway ‘home’.. Trends Immunol.

[5] Kelsoe G (1996). Life and death in germinal centers (redux).. Immunity.

[6] McHeyzer-Williams LJ, Driver DJ, McHeyzer-Williams MG (2001). Germinal center reaction.. Curr Opin Hematol.

[7] Shapiro-Shelef M, Calame K (2005). Regulation of plasma-cell development.. Nat Rev Immunol.

[8] Cerutti A, Rescigno M (2008). The biology of intestinal immunoglobulin A responses.. Immunity.

[9] Wright MD, Tomlinson MG (1994). The ins and outs of the transmembrane 4 superfamily.. Immunol Today.

[10] Berditchevski F (2001). Complexes of tetraspanins with integrins: more than meets the eye.. J Cell Sci.

[11] Tarrant JM, Robb L, van Spriel AB, Wright MD (2003). Tetraspanins: molecular organisers of the leukocyte surface.. Trends Immunol.

[12] Hemler ME (2005). Tetraspanin functions and associated microdomains.. Nat Rev Mol Cell Biol.

[13] Wright MD, Moseley GW, van Spriel AB (2004). Tetraspanin microdomains in immune cell signalling and malignant disease.. Tissue Antigens.

[14] Knobeloch KP, Wright MD, Ochsenbein AF, Liesenfeld O, Lohler J (2000). Targeted inactivation of the tetraspanin CD37 impairs T-cell-dependent B-cell response under suboptimal costimulatory conditions.. Mol Cell Biol.

[15] van Spriel AB, Puls KL, Sofi M, Pouniotis D, Hochrein H (2004). A regulatory role for CD37 in T cell proliferation.. J Immunol.

[16] Sheng KC, van Spriel AB, Gartlan KH, Sofi M, Apostolopoulos V (2008). Tetraspanins CD37 and CD151 differentially regulate Ag presentation and T-cell co-stimulation by DC.. Eur J Immunol.

[17] Meyer-Wentrup F, Figdor CG, Ansems M, Brossart P, Wright MD (2007). Dectin-1 interaction with tetraspanin CD37 inhibits IL-6 production.. J Immunol.

[18] Ariizumi K, Shen GL, Shikano S, Xu S, Ritter R (2000). Identification of a novel, dendritic cell-associated molecule, dectin-1, by subtractive cDNA cloning.. J Biol Chem.

[19] Brown GD (2006). Dectin-1: a signalling non-TLR pattern-recognition receptor.. Nat Rev Immunol.

[20] Smith KG, Light A, Nossal GJ, Tarlinton DM (1997). The extent of affinity maturation differs between the memory and antibody-forming cell compartments in the primary immune response.. Embo J.

[21] Kunkel EJ, Butcher EC (2003). Plasma-cell homing.. Nat Rev Immunol.

[22] Beagley KW, Bao S, Ramsay AJ, Eldridge JH, Husband AJ (1995). IgA production by peritoneal cavity B cells is IL-6 independent: implications for intestinal IgA responses.. Eur J Immunol.

[23] Kopf M, Baumann H, Freer G, Freudenberg M, Lamers M (1994). Impaired immune and acute-phase responses in interleukin-6-deficient mice.. Nature.

[24] Kopf M, Herren S, Wiles MV, Pepys MB, Kosco-Vilbois MH (1998). Interleukin 6 influences germinal center development and antibody production via a contribution of C3 complement component.. J Exp Med.

[25] Romani L, Mencacci A, Cenci E, Spaccapelo R, Toniatti C (1996). Impaired neutrophil response and CD4+ T helper cell 1 development in interleukin 6-deficient mice infected with Candida albicans.. J Exp Med.

[26] Bromander AK, Ekman L, Kopf M, Nedrud JG, Lycke NY (1996). IL-6-deficient mice exhibit normal mucosal IgA responses to local immunizations and Helicobacter felis infection.. J Immunol.

[27] Lopez-Mejias R, Martinez A, Del Pozo N, Fernandez-Arquero M, Ferreira A (2008). Interleukin-6 gene variation in Spanish patients with immunoglobulin-A deficiency.. Hum Immunol.

[28] Taylor PR, Tsoni SV, Willment JA, Dennehy KM, Rosas M (2007). Dectin-1 is required for beta-glucan recognition and control of fungal infection.. Nat Immunol.

[29] Saijo S, Fujikado N, Furuta T, Chung SH, Kotaki H (2007). Dectin-1 is required for host defense against Pneumocystis carinii but not against Candida albicans.. Nat Immunol.

[30] Gudlaugsson O, Gillespie S, Lee K, Vande Berg J, Hu J (2003). Attributable mortality of nosocomial candidemia, revisited.. Clin Infect Dis.

[31] Netea MG, Brown GD, Kullberg BJ, Gow NA (2008). An integrated model of the recognition of Candida albicans by the innate immune system.. Nat Rev Microbiol.

[32] DiNubile MJ, Hille D, Sable CA, Kartsonis NA (2005). Invasive candidiasis in cancer patients: observations from a randomized clinical trial.. J Infect.

[33] van Spriel AB (2003). Novel immunotherapeutic strategies for invasive fungal disease.. Curr Drug Targets Cardiovasc Haematol Disord.

[34] Koh AY, Kohler JR, Coggshall KT, Van Rooijen N, Pier GB (2008). Mucosal damage and neutropenia are required for Candida albicans dissemination.. PLoS Pathog.

[35] Korn T, Bettelli E, Gao W, Awasthi A, Jager A (2007). IL-21 initiates an alternative pathway to induce proinflammatory T(H)17 cells.. Nature.

[36] Huang W, Na L, Fidel PL, Schwarzenberger P (2004). Requirement of interleukin-17A for systemic anti-Candida albicans host defense in mice.. J Infect Dis.

[37] LeibundGut-Landmann S, Gross O, Robinson MJ, Osorio F, Slack EC (2007). Syk- and CARD9-dependent coupling of innate immunity to the induction of T helper cells that produce interleukin 17.. Nat Immunol.

[38] Zelante T, De Luca A, Bonifazi P, Montagnoli C, Bozza S (2007). IL-23 and the Th17 pathway promote inflammation and impair antifungal immune resistance.. Eur J Immunol.

[39] Bozza S, Zelante T, Moretti S, Bonifazi P, DeLuca A (2008). Lack of Toll IL-1R8 exacerbates Th17 cell responses in fungal infection.. J Immunol.

[40] Weisbart RH, Kacena A, Schuh A, Golde DW (1988). GM-CSF induces human neutrophil IgA-mediated phagocytosis by an IgA Fc receptor activation mechanism.. Nature.

[41] van Spriel AB, van den Herik-Oudijk IE, van Sorge NM, Vile HA, van Strijp JA (1999). Effective phagocytosis and killing of Candida albicans via targeting FcgammaRI (CD64) or FcalphaRI (CD89) on neutrophils.. J Infect Dis.

[42] Shibuya A, Sakamoto N, Shimizu Y, Shibuya K, Osawa M (2000). Fc alpha/mu receptor mediates endocytosis of IgM-coated microbes.. Nat Immunol.

[43] Casadevall A, Feldmesser M, Pirofski LA (2002). Induced humoral immunity and vaccination against major human fungal pathogens.. Curr Opin Microbiol.

[44] Wagner RD, Vazquez-Torres A, Jones-Carson J, Warner T, Balish E (1996). B cell knockout mice are resistant to mucosal and systemic candidiasis of endogenous origin but susceptible to experimental systemic candidiasis.. J Infect Dis.

[45] Kitamura D, Roes J, Kuhn R, Rajewsky K (1991). A B cell-deficient mouse by targeted disruption of the membrane exon of the immunoglobulin mu chain gene.. Nature.

[46] Lalor PA, Nossal GJ, Sanderson RD, McHeyzer-Williams MG (1992). Functional and molecular characterization of single, (4-hydroxy-3-nitrophenyl)acetyl (NP)-specific, IgG1+ B cells from antibody-secreting and memory B cell pathways in the C57BL/6 immune response to NP.. Eur J Immunol.

[47] Huntington ND, Xu Y, Puthalakath H, Light A, Willis SN (2006). CD45 links the B cell receptor with cell survival and is required for the persistence of germinal centers.. Nat Immunol.

[48] Lehrer RI, Cline MJ (1969). Interaction of Candida albicans with human leukocytes and serum.. J Bacteriol.

[49] Ferwerda G, Meyer-Wentrup F, Kullberg BJ, Netea MG, Adema GJ (2008). Dectin-1 synergizes with TLR2 and TLR4 for cytokine production in human primary monocytes and macrophages.. Cell Microbiol.

