# Supplementary figures and images for: The Tetraspanin Protein CD37 Regulates IgA Responses and Anti-Fungal Immunity
Source: PLoS Pathog. 2009 Mar 13;5(3):e1000338. doi: 10.1371/journal.ppat.1000338 (PMC2650281; doi:10.1371/journal.ppat.1000338)

**Figure S1.**

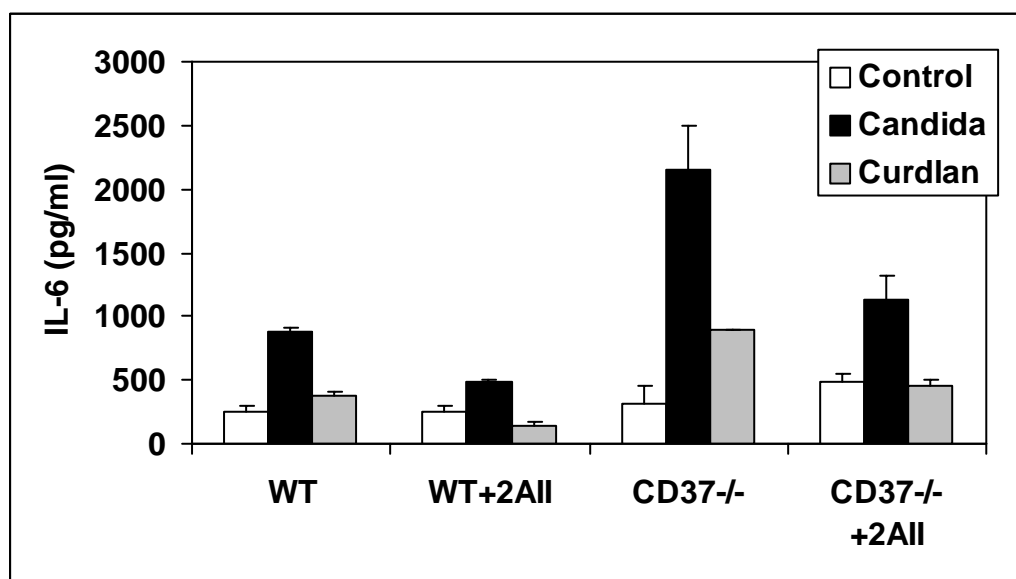

Supplement: Figure S1 — IL-6 production by WT and CD37−/− splenocytes stimulated by C. albicans is dependent on dectin-1. Spleens were removed from CD37−/− and WT mice 7 d after infection (1×10^5^ CFU C. albicans) and restimulated with heat-killed C. albicans (E∶T ratio 2∶1) or the dectin-1 ligand Curdlan (100 µg/ml) for 48 h, after which IL-6 in supernatants were measured by ELISA. Antibody 2A11 was added during stimulations (10 µg/ml) to block dectin-1. (0.01 MB PDF) [file ppat.1000338.s001.pdf]

Figure S2.

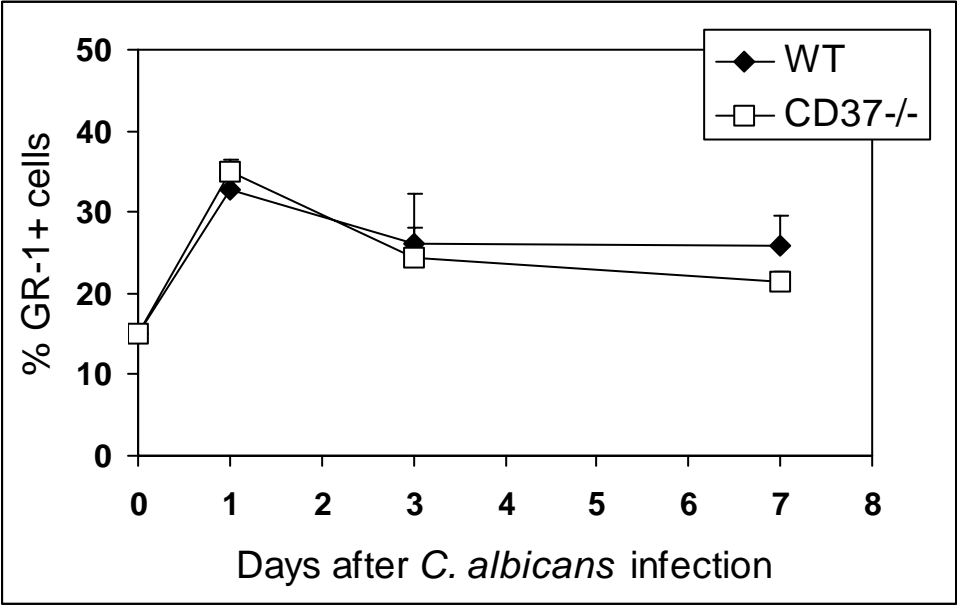

Supplement: Figure S2 — Normal increase of GR-1-positive cells in blood of CD37−/− mice. Granulopoiesis in WT and CD37−/− mice (n = 5) systemically infected with 1×10^5^ CFU C. albicans. Percentage of GR-1-positive cells in blood was determined by flow cytometry at different time points after infection. (0.01 MB PDF) [file ppat.1000338.s002.pdf]
